# The Association of Shared Care Networks With 30-Day Heart Failure Excessive Hospital Readmissions: Longitudinal Observational Study

**DOI:** 10.2196/30777

**Published:** 2022-04-06

**Authors:** Diego Pinheiro, Ryan Hartman, Jing Mai, Erick Romero, Mohammad Soroya, Carmelo Bastos-Filho, Ricardo de Carvalho Lima, Michael Gibson, Imo Ebong, Julie Bidwell, Miriam Nuno, Martin Cadeiras

**Affiliations:** 1 Unicap-Icam International School Universidade Católica de Pernambuco Recife Brazil; 2 see Acknowledgments; 3 Department of Internal Medicine Division of Cardiovascular Medicine University of California, Davis Sacramento, CA United States; 4 Polytechnic School of Pernambuco University of Pernambuco Recife Brazil; 5 Division of Cardiovascular Surgery University of Pernambuco Recife Brazil; 6 Family Caregiving Institute Betty Irene Moore School of Nursing University of California, Davis Sacramento, CA United States; 7 Department of Public Health Sciences Division of Biostatistics University of California, Davis Sacramento, CA United States

**Keywords:** patient readmission, quality assurance, health care, catchment area, health, community networks, regional medical programs

## Abstract

**Background:**

Higher-than-expected heart failure (HF) readmissions affect half of US hospitals every year. The Hospital Reduction Readmission Program has reduced risk-adjusted readmissions, but it has also produced unintended consequences. Shared care models have been advocated for HF care, but the association of shared care networks with HF readmissions has never been investigated.

**Objective:**

This study aims to evaluate the association of shared care networks with 30-day HF excessive readmission rates using a longitudinal observational study.

**Methods:**

We curated publicly available data on hospital discharges and HF excessive readmission ratios from hospitals in California between 2012 and 2017. Shared care areas were delineated as data-driven units of care coordination emerging from discharge networks. The localization index, the proportion of patients who reside in the same shared care area in which they are admitted, was calculated by year. Generalized estimating equations were used to evaluate the association between the localization index and the excessive readmission ratio of hospitals controlling for race/ethnicity and socioeconomic factors.

**Results:**

A total of 300 hospitals in California in a 6-year period were included. The HF excessive readmission ratio was negatively associated with the adjusted localization index (β=–.0474, 95% CI –0.082 to –0.013). The percentage of Black residents within the shared care areas was the only statistically significant covariate (β=.4128, 95% CI 0.302 to 0.524).

**Conclusions:**

Higher-than-expected HF readmissions were associated with shared care networks. Control mechanisms such as the Hospital Reduction Readmission Program may need to characterize and reward shared care to guide hospitals toward a more organized HF care system.

## Introduction

Higher-than-expected heart failure (HF) readmission impacts approximately half of US hospitals every year, and almost every hospital has experienced it at least once in the period between 2012 and 2017. By 2030, HF is projected to affect at least 8 million people in the United States, with an incidence of 21 per 1000 people older than 65 years and an estimated cost of US $69.8 billion [1]. The number of patients with HF receiving HF care and requiring advanced HF therapies such as left ventricular assisted devices (LVADs) will increase exponentially [2]. Addressing higher-than-expected HF readmissions for patients with HF is needed as demand increases, with the aging population requiring improved care coordination mechanisms that promote a more organized HF care system [3].

HF is managed through a complex system that serves both affluent and vulnerable patient populations, and encompasses nonlinear interactions among primary care, general cardiology, specialized HF clinics, and tertiary and quaternary centers. The implementation of any control mechanism can produce unintended consequences if the complexity of the HF care system is not taken into consideration [4,5]. Systemwide control programs such as the Hospital Reduction Readmission Program (HRRP) [6] may be a first step toward organizing the HF care system. Nonetheless, they will continue to create unintended consequences and penalize hospitals for factors beyond their control [7] unless these programs specifically foster care coordination mechanisms capable of promoting organization for HF care’s complex system.

Shared care integrates primary, secondary, and tertiary levels of care [8], and has been advocated as a necessary model to promote a more organized HF care system [9] such as the spoke-hub-and-node model [10]. Shared care has been studied among chronic diseases [11], including HF [12], but only recently has it been advocated for by international working groups as a way to organize HF care [9], particularly among patients with advanced HF [10] such as patients with LVAD support [13]. Shared care areas (SCAs) are data-driven units of care coordination captured from large-scale data on hospital discharges to patient residencies, and SCAs may explain variation in medical adherence to HF guideline-directed medical therapy [14]. The localization index (LI) of an SCA is the proportion of patients who reside in the same SCA they are admitted and is a measure of local care coordination commonly used to evaluate SCAs [15]. This study aims to evaluate the longitudinal association between higher-than-expected HF readmissions and the LI of SCAs both unadjusted and adjusted for racial/ethnic and socioeconomic factors.

## Methods

This methods section was written according to the STROBE (Strengthening the Reporting of Observational Studies in Epidemiology) standard of writing.

### Study Design, Study Setting, and Participants

This is an observational longitudinal study. All data used in this study are made publicly available by the HRRP and Office of Statewide Health Planning and Development (OSHPD). The study setting was hospitals in California during the period from 2012 to 2017. Participants were all in hospitals reported in the HRRP [6]. The eligibility criteria were as follows: at least 2 repeated measures of higher-than-expected HF readmission in the HRRP and availability of discharge data from the OSHPD [16]. These criteria enabled carrying out a longitudinal study that requires repeated measures and linking data from the HRRP with date from OSHPD. Between 233 and 237 hospitals in California were included depending on the year. Ethical approval was unnecessary because all data were at the hospital level and already made publicly available from both HRRP and OSHPD. All code, processed data, built networks, and data analysis resulting from this study are available on the Open Science Framework repository for this study [17].

### Study Outcome

The main study outcome was hospital excessive readmission ratio (ERR), which is a risk-standardized 30-day readmission ratio that adjusts for a set of patient-specific covariates such as congestive HF, renal failure, and chronic obstructive pulmonary disease [18]. It is used by the HRRP to assess excess hospital readmissions and calculate hospital penalties [6]. The ERR is calculated by dividing the predicted readmissions by the expected readmissions. Using a hierarchical generalized linear model, both predicted and expected readmissions are estimated using an adjusted average intercept over all hospitals, but predicted readmissions, in addition, are estimated using a hospital-specific intercept deviation from the adjusted average intercept over all hospitals. Such methodology, well documented in the Condition-Specific Readmission Measures Updates and Specifications Report from the Centers for Medicare & Medicaid Services [18], makes the ERR an appropriate instrument for comparing hospitals within and between years.

### Study Variables

The main study variable was the LI, which represents the proportion of patient discharges from hospitals within the same SCA of which these patients live [19,20]. A higher LI represents a homogenous SCA with localized care coordination (ie, patients tend to receive care where they live). Other study variables were the proportions of residents who were Black, Hispanic, had poverty status, and had private insurance as determined by the American Community Survey [21].

### Data Sources

The ERR data used in this study was made publicly available from the HRRP [6]. The ERR data of each year in the period from 2012 to 2017 (ie, fiscal year 2014 and 2019) was separately downloaded from HRRP and compiled into a single file. The Patient Origin/Market Share data was made publicly available from the OSHPD [16]. Patient Origin/Market Share data are aggregated numbers of emergency department (ED) discharges among zip codes of hospitals and patient residencies. Zip Codes were converted to the Zip Code Tabulation Areas (ZCTAs) using the Zip Code to ZCTA Crosswalk made publicly available by the Uniform Data System [22]. Demographic data was gathered for the state of California from the American Community Survey [21].

### Uncovering Shared Care Areas and Localization Index From Hospital-Patient Discharge Data

Six yearly hospital-patient discharge networks were built from OSHPD hospital-patient ED discharges between 2012 to 2017. In a hospital-patient discharge network [15], a node is the ZCTA of a hospital or patient residency, and the link between two nodes (ie, ZCTAs) is the total number of ED discharges. For each yearly hospital-patient discharge network, SCAs were delineated using community detection algorithms. Each delineated SCA consists of a set of ZCTAs in which hospitals are embedded. A set of four diverse community detection algorithms were considered to decrease both variability and bias [23]. The algorithms were Louvain [24] with resolution equal to 1, Stochastic Block Model [25,26] with degree corrected, Infomap [27] with two levels, and Speaker-Listener Label Propagation [28] with postprocessing threshold equal to 0.5

### Statistical Analysis

The ERR hospitals and the LI of SCAs were integrated at each year by linking the ZCTAs of hospitals and SCAs (Table S1 and Figure S1 in Multimedia Appendix 1). A longitudinal regression was specified in which the dependent variable ERR of a hospital at time *t* as a function of the LI of its SCA at time *t*. We used a generalized estimating equation (GEE) using a Gaussian family and an exchangeable working correlation structure to account for multiple observations of ERR from the same hospital across years and SCAs [29]. The estimated regression coefficients (beta) were used to measure unadjusted associations between the dependent and independent variables, and adjusted associations after controlling for racial/ethnic and socioeconomic confounders associated with HF readmission at the regional level [30]. The GEE was estimated using the Statsmodels Python package [31]. Additionally, hospitals were stratified based on quartiles of the LI and all covariates that were found statistically significant, and median values of ERRs and percentage of hospitals penalized were calculated for each quartile (Q1, Q2, Q3, Q4). We estimated 95% CIs using 10,000 bootstrap samples with replacement from each quartile, the estimation of CIs for medians using the Bootstrapped Python package [32].

### Predicting Higher-Than-Expected Heart Failure Readmissions for Changes in Localization Index

The estimated GEE model was used to predict HF’s ERRs assuming a range of changes in the LI in SCAs with distinct percentages of Black residents, the only statistically significant covariate. The differences in the LI between subsequent years were calculated for all hospitals. The 25th, 50th, and 75th percentiles were separately calculated for both positive (+q1, +q2, and +q3) and negative (–q1, –q2, and –q3) differences. The SCAs were stratified by quartiles of Black residents (Q1, Q2, Q3, and Q4). The ERR was predicted using the GEE model after each positive and negative percentile difference in the LI was applied to the stratified SCA data.

## Results

### Descriptive Statistics of Heart Failure Hospital Readmissions in the United States and California

The ERR is calculated every year by the HRRP for the approximately 2700 to 2900 hospitals in the United States, from which 233 to 237 hospitals are from California (Table 1). Overall, approximately half of US hospitals are penalized, and this percentage has not changed during the study period between 2012 to 2017. The ERR (and the percentage of hospitals penalized) of US hospitals have remained approximately constant during the study period, from 1.0013 (49.76%) in 2012 to 1.0016 (48.94%) in 2017. The ERR (and the percentage of hospitals penalized) of hospitals in California increased from 0.9914 (49.36%) to 1.0087 (56.12%). In 2017, the percentage of hospitals penalized in California (56.12%, 95% CI 49.75%-62.29%) is slightly higher than that among all hospitals in the United States (48.91%, 95% CI 47.06%-50.76%). Although not statistically significant, the ERR SD appears to be decreasing over the years.

**Table 1:** Descriptive statistics of excessive readmission ratio (ERR) and percentage of hospitals penalized in the United States and California.

Region		2012	2013	2014	2015	2016	2017
**United States**							
	Hospitals, n	2864	2860	2825	2820	2827	2793
	Hospitals penalized (%)	49.76	48.95	49.17	49.22	49.45	48.94
	ERR	1.0013	1.0012	1.0010	1.0012	1.0018	1.0016
	ERR SD	0.0844	0.0809	0.0803	0.0774	0.0776	0.0753
**California**							
	Hospitals, n	233	233	233	233	237	237
	Hospitals penalized (%)	49.36	48.50	56.22	55.79	51.90	56.12
	ERR	0.9914	0.9963	1.0034	1.0057	1.0049	1.0087
	ERR SD	0.0761	0.0778	0.0760	0.0731	0.0720	0.0703

### Association of the Excessive Readmission Ratio and Localization Index

**Figure 1:**
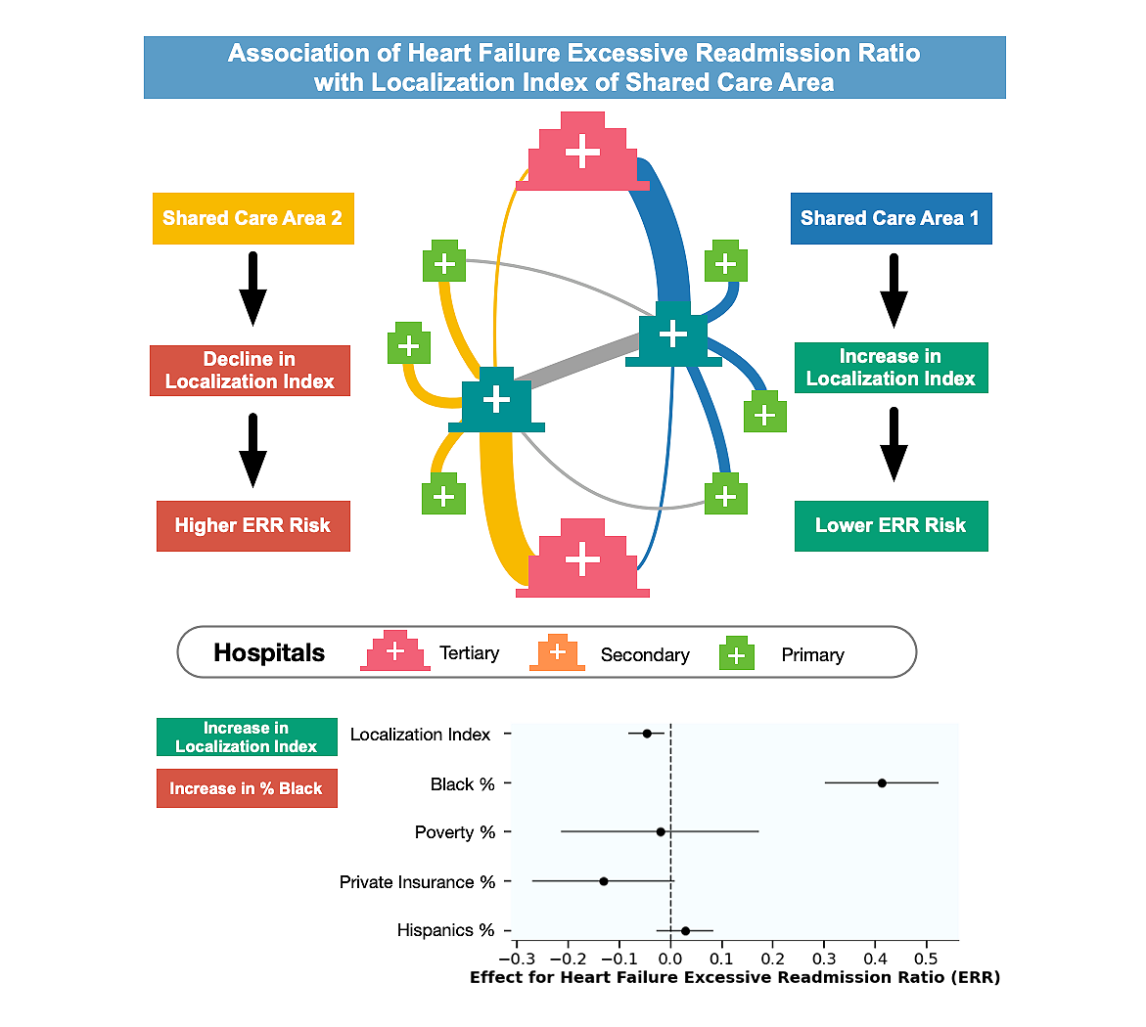
Central illustration: association of heart failure excessive readmission with shared care networks. Hospitals are embedded in shared care areas (SCAs), which are data-driven units of care coordination emerging from the discharge networks among hospitals. The localization index is the proportion of patient discharges from hospitals within the same SCA in which these patients live. The heart failure ERRs of hospitals are associated with the SCA localization index in which they are embedded.

The results of the quartile analysis indicate that the ERR of hospitals was negatively associated with the LI (Table 2) as well as with the percentage of Black residents (Table 3). In 2017, for instance, the ERR of hospitals in SCAs with the lowest quartile (Q1) of the LI was 1.03 (95% CI 1.02-1.04) with 65.7% (95% CI 59.4%-72.0%) of hospitals penalized. In SCAs with the highest quartile (Q4) of the LI; however, the median ERR was 0.98 (95% CI 0.97-0.99) with only 43.1% (95% CI 35.3%-51.0%) of hospitals penalized. From 2012 to 2017, the disparities between the ERR and percentage of hospitals penalized among SCAs belonging to the lowest (Q1) and highest LI (Q4) quartiles has increased mainly because of increases in the ERR and percentage of hospitals penalized within SCAs in the lowest LI quartile (Q1). Similarly, in 2017, the ERR of hospitals in SCAs with the lowest quartile (Q1) of Black residents was 0.99 (95% CI 0.98-1.0) with 45.2% (95% CI 38.2%-52.2%) of hospitals penalized. In SCAs with the highest percentage of Black residents quartile (Q4), however, the median ERR was 1.03 (95% CI 1.02-1.04) with 67.6% (95% CI 60.7%-74.6%) of hospitals penalized. The percentage of Black residents is slightly higher in SCAs with lower localization (Table S4 in Multimedia Appendix 1). The results of the regression analysis (Figure 1 and Table 4) indicate that the ERR of hospitals was negatively associated with the adjusted and unadjusted LI of their SCAs (eg, ERRs were lower when hospitals were located in SCAs where more patients received care close to where they resided) according to both unadjusted (β=–.0717; *P*<.001) and adjusted (β=–.0495; *P*=.049) coefficients when the regression was controlled for racial/ethnic and socioeconomic covariates. The percentage of Black residents in the SCA was the only covariate with a statistically significant association according to the regression coefficient (β=.3892; *P*<.001). The results can be separately analyzed for each community detection algorithm (Table S3, Multimedia Appendix 1), and the Stochastic Block Model uncovered SCAs with the LI anomalously lower and was not considered in the final analysis. The results can be separately analyzed for each community detection algorithm for ERR (Table S5 in Multimedia Appendix 1), percentage of hospitals penalized (Table S6 in Multimedia Appendix 1), and the percentage of Black residents (Table S7 in Multimedia Appendix 1).

**Table 2:** Excessive readmission ratios (ERRs) for hospitals in California by the localization index (LI) quartile.

LI^a^		2012 (95% CI)	2013 (95% CI)	2014 (95% CI)	2015 (95% CI)	2016 (95% CI)	2017 (95% CI)
**ERR^b^**							
	Q1	1.0 (0.99-1.01)	1.0 (0.99-1.01)	1.01 (1.0-1.02)	1.02 (1.01-1.03)	1.02 (1.01-1.03)	1.03 (1.02-1.04)
	Q2	1.0 (0.99-1.01)	1.01 (1.0-1.02)	1.02 (1.01-1.03)	1.02 (1.01-1.03)	1.01 (1.0-1.02)	1.01 (1.0-1.02)
	Q3	0.99 (0.97-1.0)	1.0 (0.98-1.01)	0.99 (0.98-1.0)	1.0 (0.99-1.0)	0.99 (0.98-1.0)	1.0 (0.99-1.02)
	Q4	0.98 (0.97-0.99)	0.98 (0.97-0.99)	0.99 (0.98-1.0)	0.99 (0.98-1.0)	0.99 (0.98-1.0)	0.98 (0.97-0.99)
**Hospitals penalized (%)**							
	Q1	53.24 (45.61-60.82)	50.58 (43.02-58.14)	62.09 (54.6-68.97)	67.0 (59.66-73.86)	60.63 (53.88-67.78)	65.69 (59.42-71.98)
	Q2	53.13 (46.39-60.31)	52.75 (45.34-60.25)	67.07 (59.63-74.53)	58.85 (51.27-66.46)	54.1 (47.03-61.08)	58.17 (50.85-65.54)
	Q3	45.02 (37.32-52.82)	50.82 (43.65-58.01)	49.48 (42.39-56.52)	51.79 (44.67-58.88)	48.68 (41.53-55.74)	54.00 (46.55-61.49)
	Q4	45.32 (38.54-52.6)	40.53 (33.51-47.57)	47.78 (40.56-55.0)	45.79 (38.1-53.57)	43.61 (36.2-51.53)	43.14 (35.29-50.98)

^a^CIs estimated by 10,000 bootstrap samples with replacement.

^b^Quartiles Q1 (0-25th), Q2 (25th-50th), Q3 (50th-75th), and Q4 (75th-100th).

**Table 3:** Excessive readmission ratios (ERRs) for hospitals in California by percentage of Black residents in the shared care area.

LI^a,b^			2012 (95% CI)		2013 (95% CI)		2014 (95% CI)		2015 (95% CI)		2016 (95% CI)		2017 (95% CI)
**ERR^c^**													
	Q1	0.96 (0.95-0.97)		0.97 (0.96-0.98)		0.97 (0.96-0.98)		0.98 (0.97-0.99)		0.98 (0.97-0.99)		0.99 (0.98-1.0)	
	Q2	0.99 (0.98-1.0)		0.99 (0.98-1.01)		1.0 (0.98-1.01)		1.0 (0.98-1.01)		1.0 (0.99-1.01)		1.0 (0.99-1.02)	
	Q3	1.0 (0.99-1.01)		1.0 (0.99-1.01)		1.02 (1.01-1.03)		1.02 (1.01-1.03)		1.01 (1.0-1.02)		1.01 (1.0-1.02)	
	Q4	1.02 (1.01-1.03)		1.02 (1.01-1.03)		1.03 (1.02-1.04)		1.04 (1.03-1.05)		1.03 (1.02-1.04)		1.03 (1.02-1.04)	
**Hospitals penalized (%)**													
	Q1	33.34 (26.11-40.56)		36.65 (29.44-43.89)		36.65 (29.44-43.89)		33.89 (27.22-40.56)		38.13 (31.18-45.16)		45.17 (38.17-52.15)	
	Q2	50.82 (43.24-57.84)		48.09 (41.08-55.14)		50.85 (43.78-57.84)		54.57 (47.03-61.62)		52.48 (45.41-59.46)		52.99 (45.95-60.0)	
	Q3	53.05 (45.73-60.98)		55.49 (47.56-63.41)		65.84 (58.54-73.17)		68.28 (60.98-75.0)		59.94 (52.69-67.07)		59.9 (52.69-67.07)	
	Q4	61.14 (53.53-68.24)		54.69 (47.06-61.78)		73.47 (66.47-80.0)		68.22 (61.18-75.29)		58.42 (50.87-65.9)		67.64 (60.69-74.57)	

^a^LI: localization index.

^b^CIs estimated by 10,000 bootstrap samples with replacement.

^c^Quartiles Q1 (0-25th), Q2 (25th-50th), Q3 (50th-75th), and Q4 (75th-100th).

**Table 4:** Results of the generalized estimating equations regression for excessive readmission ratios.

Estimator		Coefficient (SE)	*z*	*P* value
**Unadjusted model**				
	Intercept	1.0733 (0.014)	75.626	<.001
	Localization index	–0.0722 (0.0170)	–4.2190	<.001
**Adjusted model**				
	Intercept	1.1054 (0.067)	16.558	<.001
	Localization index	–0.0474 (0.0180)	–2.6670	.008
	% Black	0.4128 (0.0570)	7.2970	<.001
	% poverty	–0.0208 (0.0990)	–0.2100	.83
	% private insurance	–0.1317 (0.0710)	–1.8500	.06
	% Hispanic	0.0278 (0.0290)	0.9710	.33

### Predictions of Excessive Readmission Ratio Based on Changes in Localization Index

**Figure 2:**
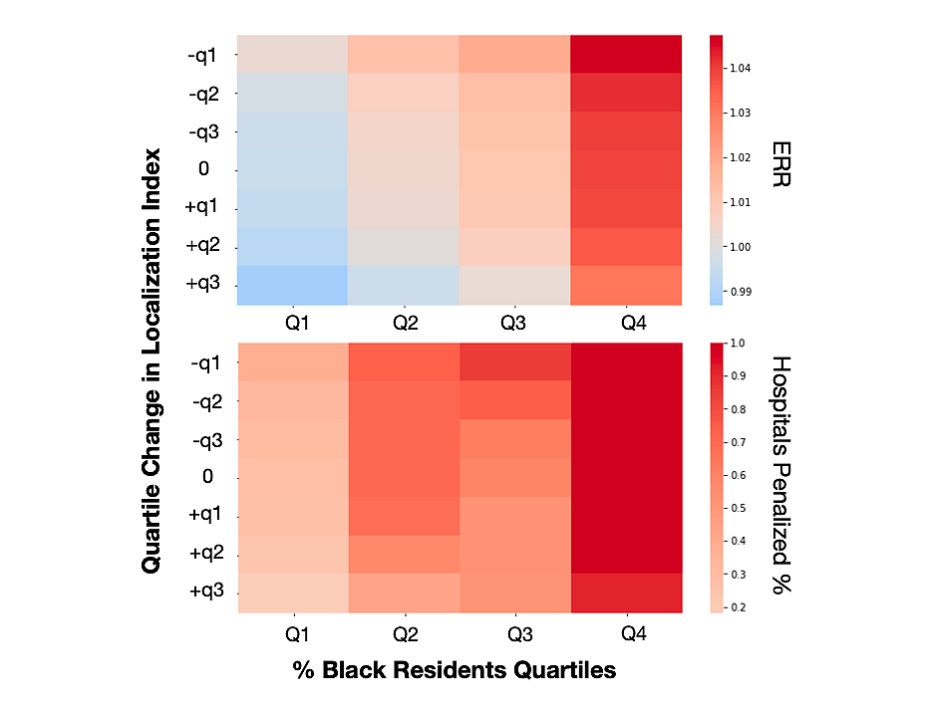
Predictions of ERRs and percentage of hospitals penalized based on changes in localization index. The heart failure ERRs of hospitals are negatively associated with the localization index of the shared care areas (SCAs) in which they are embedded and positively associated with the percentage of Black residents within the SCA. The percentage of Black residents in SCAs were stratified into four quartiles: Q1 0.20%-1.96%, Q2 1.96%-4.16%, Q3 4.16%-7.85%, Q4 7.85%-17.6%. The quartiles in localization index differences were separately calculated for negative (–q1, –q2, –q3)=(–0.167, –0.058, –0.015) and positive (+q1, +q2, +q3)=(0.019, 0.070, 0.179) of localization index differences. ERR: excessive readmission ratio.

The predictions of ERRs and percentage of hospitals penalized based on changes in the LI (Table 5 and Figure 2) demonstrated the negative association with the LI of their SCAs as well as the positive association with the percentage of Black residents in the SCAs. The percentage range of Black residents in the stratified SCAs were 0.20% to 1.96% in Q1, 1.96% to 4.16% in Q2, 4.16% to 7.85% in Q3, and 7.85% to 17.6% in Q4. The quartiles in the LI for negative differences were –0.167 (–q3), –0.058 (–q2), and –0.015 (–q1); positive differences were 0.019 (+q1), 0.070 (+q2), and 0.179 (+q3). In Q1 and Q4, the estimated median ERR was 0.995 (95% CI 0.994-0.996) and 1.039 (95% CI 1.038-1.041), respectively, with 27.5% (95% CI 24.6%-30.4%) and 100% (95% CI 100%-100%) of hospitals penalized, respectively. If the LI decreases by –0.167 (ie, a –q3 LI change), the median ERR is predicted at 1.003 (95% CI 1.002-1.004) and 1.047 (95% CI 1.046-1.048) in Q1 and Q4, respectively, with 39.2% (95% CI 35.8%-42.4%) and 100% (95% CI 100%-100%) of hospitals penalized. Conversely, if the LI increases by 0.179 (ie, a +q4 LI change), the median ERR is predicted at 0.987 (95% CI 0.986-0.988) and 1.031 (95% CI 1.030-1.032) in Q1 and Q4, respectively, with 18.1% (95% CI 15.6%-20.8%) and 91.6% (95% CI 89.7%-93.4%) of hospitals penalized.

**Table 5:** Predictions of excessive readmission ratios (ERRs) and percentage of hospitals penalized based on changes in localization index (LI).

Change in LI^a^			% Black (Q1; 95% CI)^b^		% Black (Q2; 95% CI)^b^		% Black (Q3; 95% CI)^b^		% Black (Q4; 95% CI)^b^
**ERR**									
	–q3	1.003 (1.002-1.004)		1.012 (1.011-1.014)		1.019 (1.018-1.02)		1.047 (1.046-1.048)	
	–q2	0.998 (0.997-0.999)		1.007 (1.006-1.008)		1.014 (1.013-1.015)		1.042 (1.041-1.043)	
	–q1	0.996 (0.995-0.997)		1.005 (1.004-1.006)		1.012 (1.011-1.013)		1.04 (1.039-1.041)	
	0	0.995 (0.994-0.996)		1.004 (1.003-1.006)		1.011 (1.01-1.012)		1.039 (1.038-1.041)	
	+q1	0.994 (0.993-0.995)		1.003 (1.002-1.005)		1.01 (1.009-1.011)		1.038 (1.037-1.04)	
	+q2	0.992 (0.991-0.993)		1.001 (1.0-1.002)		1.008 (1.007-1.009)		1.036 (1.035-1.037)	
	+q3	0.987 (0.986-0.988)		0.996 (0.995-0.997)		1.002 (1.001-1.004)		1.031 (1.03-1.032)	
**Hospitals penalized (%)**									
	–q3	0.392 (0.358-0.424)		0.736 (0.706-0.766)		0.856 (0.832-0.879)		1.0 (1.0-1.0)	
	–q2	0.323 (0.291-0.354)		0.707 (0.676-0.737)		0.744 (0.715-0.772)		1.0 (1.0-1.0)	
	–q1	0.299 (0.269-0.329)		0.704 (0.673-0.734)		0.624 (0.591-0.656)		1.0 (1.0-1.0)	
	0	0.275 (0.246-0.304)		0.704 (0.673-0.734)		0.592 (0.561-0.624)		1.0 (1.0-1.0)	
	+q1	0.273 (0.243-0.302)		0.686 (0.656-0.718)		0.524 (0.492-0.557)		1.0 (1.0-1.0)	
	+q2	0.242 (0.213-0.271)		0.574 (0.542-0.606)		0.525 (0.492-0.557)		1.0 (1.0-1.0)	
	+q3	0.181 (0.156-0.208)		0.432 (0.398-0.466)		0.519 (0.486-0.552)		0.916 (0.897-0.934)	

^a^Changes in LI were measured as quartiles of negative differences (–q1, –q2, –q3), positive differences (+q1, +q2, +q3), and zero (no change).

^b^The quartile of % Black residents are Q1 (0 to 25th), Q2 (25th to 50th), Q3 (50th to 75th), and Q4 (75th to 100th).

## Discussion

### Principal Findings

Regional variation in health care delivery is a ubiquitous phenomenon [3,19], and the HRRP may have differently impacted almost 3000 US hospitals depending on their state. The main finding in this study is that higher-than-expected HF hospital readmissions are associated with the share care networks in which hospitals are embedded. Specifically, hospitals within SCAs with a high LI are associated with lower ERRs than hospitals within SCAs with lower LIs. The LI represents the proportion of patient discharges from hospitals within the same SCA of which these patients live. The LI is widely used as a measure of care coordination and unwarranted health care variation [4,19], but to our knowledge, this is the first documentation of its association with HF higher-than-expected readmissions. In this study, the LI is ultimately derived from the shared care discharge networks. In SCAs with a high LI, discharges are localized with a lower proportion of discharges of patients from other SCAs. Not only has shared care been advocated as an appropriate model to organize HF care [9,10], but partnerships among community physicians and local hospitals have been identified as hospital strategies to reduce 30-day HF readmission [33]. Characterizing shared care networks provides a road map for hospitals to work together, improving their shared care network as a whole instead of focusing on their hospital penalties.

Though the HRRP is a nationwide effort to reduce higher-than-expected hospital readmissions, it has also created unintended consequences in the complex system of HF care by penalizing hospitals for issues beyond their control, leaving them without specific guidance on how to improve and focusing on punishment instead of process improvements [7]. Patients with HF should be managed as a continuum of care within the primary, secondary, and tertiary level of care, promoting timely patient referrals and delivering care within a strong working relationship [9]. Integrated HF care will improve care coordination that influences patient outcomes. The features identified that result in improved shared care include liaisons between levels of care and institutions, shared professional education, and medication optimization. Comprehensive pathways across primary, secondary, and tertiary care and institutions should be developed and implemented considering patients and health care providers in the design of these pathways [34].

The association of ERRs with shared care networks, however, seems to vary depending on the ethnic/racial and socioeconomic composition of SCAs. In this study, ERR is positively associated with the percentage of Black residents in the SCA. Ethnic/racial disparities may contribute to HF hospital readmissions [20,30,33,35], and HF readmission rates are consistently higher for Black patients [35-37]. In a previous case-control study [30], after matching maximum penalty hospitals as cases to their nearest nonpenalty hospitals as controls, the authors found that maximum penalty hospitals were more likely than controls to be in counties with low socioeconomic status.

The regional variation on the impact of the HRRP raises the following question: how much HF higher-than-expected readmissions are related to hospital-specific performance, and how much it is related to issues beyond the control of a hospital? Additionally, the increased association of the ERR with the LI in SCAs with increasingly higher percentages of Black residents raises the following question: how can improved shared care networks reduce HF disparities among underserved and marginalized groups? Our findings will hopefully motivate cluster randomized clinical trials [38] to evaluate how improved shared care models will reduce hospital readmissions and overall costs, increase adherence to guideline-directed medical therapy, and improve clinical outcomes such as survival and development of chronic conditions.

### Limitations

The HRRP is a nationwide program, but our study only considered hospitals in California because large-scale hospital-specific discharge data at the ZCTA level is not publicly available to examine all US hospitals. Our finding only applies to higher-than-expected HF readmissions, and the generalization to conditions other than HF (eg, acute myocardial infarction, pneumonia, and chronic obstructive pulmonary disease) will require further investigation. The primary outcome used in our study, the ERR, is a ratio between two hospital-level regressions that can be used across heterogeneous hospitals but has little inherent variability. In its current version, our study neglects to model the interactions between SCAs, which deserves further investigation. Although our study assumes that the ERR can be used to compare different hospitals as it accounts for a plethora of factors associated with the hospital-level HF readmissions at the individual level, our findings should be interpreted at the hospital level.

### Conclusions

Shared care models have been advocated for in HF care but have not been explicitly characterized and rewarded by nationwide control programs such as the HRRP or health systems. In this study, we evaluated the association of higher-than-expected HF readmissions with shared care networks by curating publicly available large-scale hospital-level data on HF ERRs from Medicare HRRP as well as hospital-patient discharges from OSHPD. HF ERRs of hospitals were associated with the LI of the SCAs in which they were embedded, even after controlling for socioeconomic disparities. The HRRP, health systems, and hospitals should characterize and reward models of shared care practices for promoting the necessary integration capable of producing a sustainable and equitable HF care system. The higher-than-expected HF readmission of hospitals was associated with the shared care networks in which hospitals were embedded and the ethnic/racial composition of their SCAs. Hospitals should collectively work to systematically improve their shared care networks for improved HF care.

Improved shared care networks of HF care could mitigate higher-than-expected HF readmissions, especially among underserved and marginalized groups, and translate into economic benefits. Implementation of this model will require collaboration between providers and hospital administrations. Future clinical trials will be needed to evaluate the impact of systematic implementation of improved shared care models of HF to improve higher-than-expected HF readmissions.

## Appendix

Multimedia Appendix 1 Supplemental material.

## Abbreviations

ED: emergency department
ERR: excessive readmission ratio
GEE: generalized estimating equation
HF: heart failure
HRRP: Hospital Reduction Readmission Program
LI: localization index
LVAD: left ventricular assisted device
OSHPD: Office of Statewide Health Planning and Development
SCA: shared care area
STROBE: Strengthening the Reporting of Observational Studies in Epidemiology
ZCTA: Zip Code Tabulation Area

## Data Availability

All data used in this work is made publicly available by the Hospital Reduction Readmission Program and Office of Statewide Health Planning and Development.

## References

[1] Benjamin EJ, Muntner P, Alonso A, Bittencourt MS, Callaway CW, Carson AP, Chamberlain AM, Chang AR, Cheng S, Das SR, Delling FN, Djousse L, Elkind MS, Ferguson JF, Fornage M, Jordan LC, Khan SS, Kissela BM, Knutson KL, Kwan TW, Lackland DT, Lewis TT, Lichtman JH, Longenecker CT, Loop MS, Lutsey PL, Martin SS, Matsushita K, Moran AE, Mussolino ME, O'Flaherty M, Pandey A, Perak AM, Rosamond WD, Roth GA, Sampson UK, Satou GM, Schroeder EB, Shah SH, Spartano NL, Stokes A, Tirschwell DL, Tsao CW, Turakhia MP, VanWagner LB, Wilkins JT, Wong SS, Virani SS, American Heart Association Council on Epidemiology and Prevention Statistics Committee and Stroke Statistics Subcommittee (2019). Heart disease and stroke statistics-2019 update: a report from the American Heart Association. Circulation.

[2] Yin MY, Strege J, Gilbert EM, Stehlik J, McKellar SH, Elmer A, Anderson T, Aljuaid M, Nativi-Nicolau J, Koliopoulou AG, Davis E, Fang JC, Drakos SG, Selzman CH, Wever-Pinzon O (2020). Impact of shared care in remote areas for patients with left ventricular assist devices. JACC Heart Fail.

[3] Wennberg JE (2020). Tracking Medicine.

[4] Lipsitz LA (2012). Understanding health care as a complex system: the foundation for unintended consequences. JAMA.

[5] Sturmberg J, Lanham HJ (2014). Understanding health care delivery as a complex system: achieving best possible health outcomes for individuals and communities by focusing on interdependencies. J Eval Clin Pract.

[6] Hospital Readmissions Reduction Program (HRRP). Centers for Medicare and Medicaid Services.

[7] Psotka MA, Fonarow GC, Allen LA, Joynt Maddox KE, Fiuzat M, Heidenreich P, Hernandez AF, Konstam MA, Yancy CW, O'Connor CM (2020). The Hospital Readmissions Reduction Program: nationwide perspectives and recommendations: a JACC: heart failure position paper. JACC Heart Fail.

[8] Hickman M, Drummond N, Grimshaw J (1994). A taxonomy of shared care for chronic disease. J Public Health Med.

[9] Crespo-Leiro MG, Metra M, Lund LH, Milicic D, Costanzo MR, Filippatos G, Gustafsson F, Tsui S, Barge-Caballero E, De Jonge N, Frigerio M, Hamdan R, Hasin T, Hülsmann M, Nalbantgil S, Potena L, Bauersachs J, Gkouziouta A, Ruhparwar A, Ristic AD, Straburzynska-Migaj E, McDonagh T, Seferovic P, Ruschitzka F (2018). Advanced heart failure: a position statement of the Heart Failure Association of the European Society of Cardiology. Eur J Heart Fail.

[10] Huitema AA, Harkness K, Heckman GA, McKelvie RS (2018). The spoke-hub-and-node model of integrated heart failure care. Can J Cardiol.

[11] Smith SM, Allwright S, O'Dowd T (2008). Does sharing care across the primary-specialty interface improve outcomes in chronic disease? A systematic review. Am J Manag Care.

[12] Pearl A, Wright SP, Gamble GD, Muncaster S, Walsh HJ, Sharpe N, Doughty RN (2003). The effect of an integrated care approach for heart failure on general practice. Fam Pract.

[13] Kiernan MS, Joseph SM, Katz JN, Kilic A, Rich JD, Tallman MP, Van Buren P, Lyons JJ, Bethea B, Eckman P, Gosev I, Lee SS, Soleimani B, Takayama H, Patel CB, Uriel N, Evolving Mechanical Support Research Group (EMERG) Investigators (2015). Sharing the care of mechanical circulatory support: collaborative efforts of patients/caregivers, shared-care sites, and left ventricular assist device implanting centers. Circ Heart Fail.

[14] Yin MY, Strege J, Gilbert EM, Stehlik J, McKellar SH, Elmer A, Anderson T, Aljuaid M, Nativi-Nicolau J, Koliopoulou AG, Davis E, Fang JC, Drakos SG, Selzman CH, Wever-Pinzon O (2020). Impact of shared care in remote areas for patients with left ventricular assist devices. JACC Heart Fail.

[15] Hu Y, Wang F, Xierali IM (2018). Automated delineation of hospital service areas and hospital referral regions by modularity optimization. Health Serv Res.

[16] Patient Origin/Market Share (Pivot Profile)–inpatient, emergency department, and ambulatory surgery. California Health and Human Services Open Data.

[17] Heart failure excess readmission and shared care area data. Open Science Framework.

[18] Readmission Measures Methodology.

[19] Wennberg J, Cooper MM (1996). The Dartmouth Atlas of Health Care.

[20] Wallace DJ, Mohan D, Angus DC, Driessen JR, Seymour CM, Yealy DM, Roberts MM, Kurland KS, Kahn JM (2018). Referral regions for time-sensitive acute care conditions in the United States. Ann Emerg Med.

[21] American Community Survey. United States Census.

[22] Zip Code to ZCTA crosswalk. USD Mapper.

[23] Pinheiro D, Hartman R, Romero E, Menezes R, Cadeiras M, Barbosa H, Gomez-Gardenes J, Gonçalves B, Mangioni G, Menezes R, Oliveira M (2020). Network-based delineation of health service areas: a comparative analysis of community detection algorithms. Complex Networks XI: Proceedings of the 11th Conference on Complex Networks CompleNet 2020.

[24] Blondel VD, Guillaume J, Lambiotte R, Lefebvre E (2008). Fast unfolding of communities in large networks. J Stat Mechanics.

[25] Peixoto TP (2014). Hierarchical block structures and high-resolution model selection in large networks. Phys Rev X.

[26] Peixoto TP (2015). Model selection and hypothesis testing for large-scale network models with overlapping groups. Phys Rev X.

[27] Rosvall M, Bergstrom CT (2008). Maps of random walks on complex networks reveal community structure. Proc Natl Acad Sci U S A.

[28] Xie J, Szymanski BK, Liu X (2011). SLPA: uncovering overlapping communities in social networks via a speaker-listener interaction dynamic process.

[29] Liang K, Zeger SL (1986). Longitudinal data analysis using generalized linear models. Biometrika.

[30] Caracciolo C, Parker D, Marshall E, Brown J (2017). Excess readmission vs excess penalties: maximum readmission penalties as a function of socioeconomics and geography. J Hosp Med.

[31] statsmodels 0.13.2. The Python Package Index.

[32] bootstrapped 0.0.2. The Python Package Index.

[33] Bradley EH, Curry L, Horwitz LI, Sipsma H, Wang Y, Walsh MN, Goldmann D, White N, Piña IL, Krumholz HM (2013). Hospital strategies associated with 30-day readmission rates for patients with heart failure. Circ Cardiovasc Qual Outcomes.

[34] MacInnes J, Williams L (2018). A review of integrated heart failure care. Prim Health Care Res Dev.

[35] Chaiyachati KH, Qi M, Werner RM (2018). Changes to racial disparities in readmission rates after Medicare's Hospital Readmissions Reduction Program within safety-net and non-safety-net hospitals. JAMA Netw Open.

[36] Durstenfeld MS, Ogedegbe O, Katz SD, Park H, Blecker S (2016). Racial and ethnic differences in heart failure readmissions and mortality in a large municipal healthcare system. JACC Heart Fail.

[37] Churchwell K, Elkind MSV, Benjamin RM, Carson AP, Chang EK, Lawrence W, Mills A, Odom TM, Rodriguez CJ, Rodriguez F, Sanchez E, Sharrief AZ, Sims M, Williams O, American Heart Association (2020). Call to action: structural racism as a fundamental driver of health disparities: a presidential advisory from the American Heart Association. Circulation.

[38] Mercer T, Njuguna B, Bloomfield GS, Dick J, Finkelstein E, Kamano J, Mwangi A, Naanyu V, Pastakia SD, Valente TW, Vedanthan R, Akwanalo C (2019). Strengthening Referral Networks for Management of Hypertension Across the Health System (STRENGTHS) in western Kenya: a study protocol of a cluster randomized trial. Trials.

